# A 7‐year‐old with extravaginal torsion of an undescended testicle in the left inguinal region: The first case report from Syria

**DOI:** 10.1002/ccr3.9013

**Published:** 2024-05-27

**Authors:** Yousef Alsaffaf, Hazem Arab, Jaafar Jaafar, Mohammad Shehadeh

**Affiliations:** ^1^ Faculty of Medicine Hama University Hama Syria; ^2^ Department of Urology National Hama Hospital Hama Syria

**Keywords:** Case report, Inguinal region, Orchidectomy, Testicular gangrene, Undescended testicular torsion

## Abstract

The incidence of undescended testicles torsion in a 7‐year‐old is rare, making it a clinically unusual occurrence. Emphasizing the importance of thorough urogenital assessments in children, particularly in underserved communities, is critical to prevent serious complications like testicular gangrene.

## 1 INTRODUCTION

Undescended testicle, also known as cryptorchidism, is a testicle that sits in its development trajectory instead of in the scrotum.^1^ Undescended testicle is a common birth abnormality in children, typically diagnosed and treated before the age of 18 months.^2^, ^3^ The prevalence of undescended testicles in adults and children older than one year is 0.8%–1%.^4^ Few undescended testicle torsion cases have been reported.^2^ In our case, a 7‐year‐old child experienced torsion of an undescended testicle in the inguinal region, an unusual occurrence for individuals of that age group. There have been very few reported cases of this specific location in the previous literature.

## 2 CASE HISTORY

**FIGURE 1:**
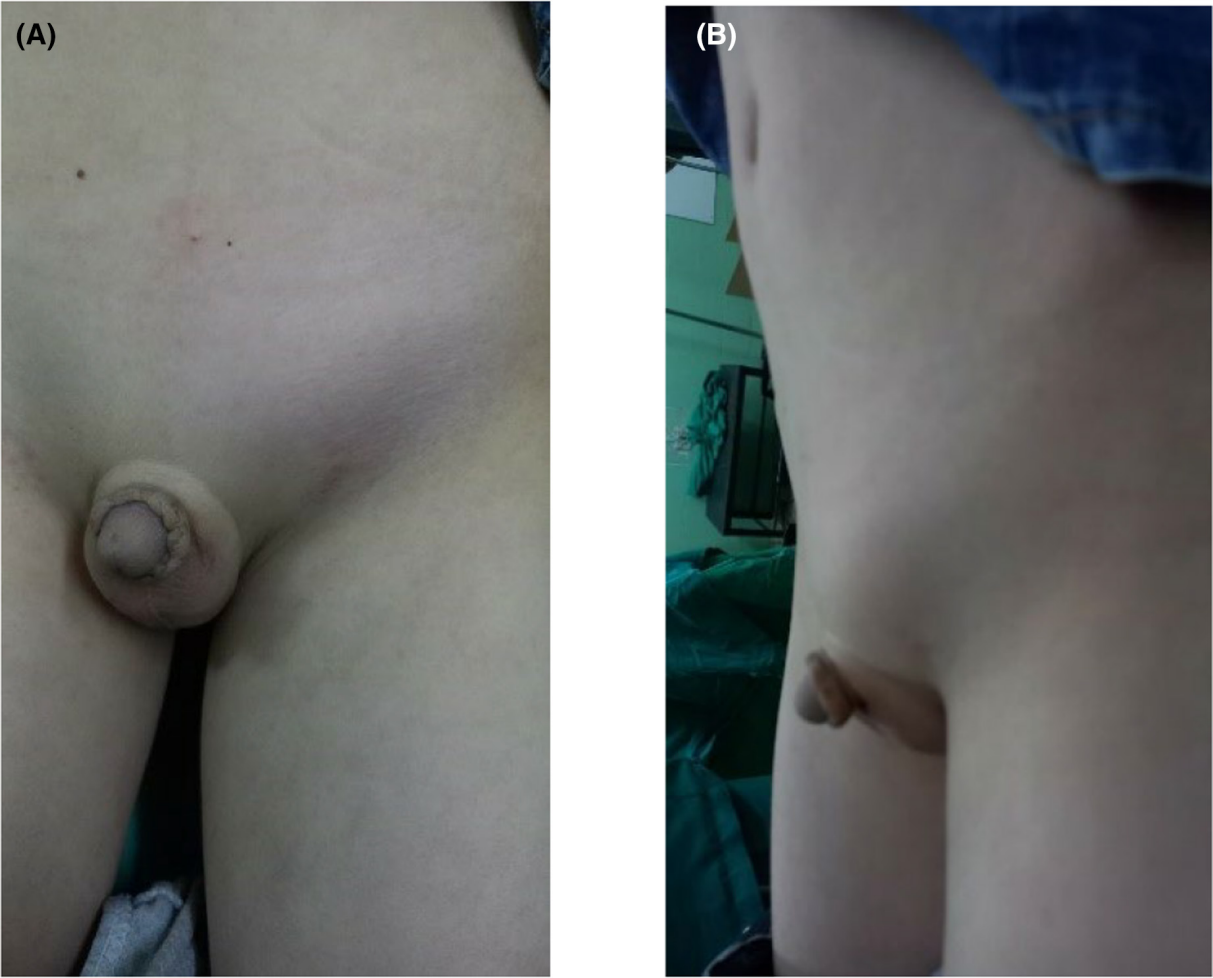
(A) Frontal view; (B) Lateral view. Physical examination revealed the left inguinal region swelling and tenderness.

A 7‐year‐old child presented to the emergency department with abdominal pain, nausea, and fever 3 days ago. The temperature was elevated, while both the patient's other vital signs and his level of awareness were normal. The patient had no prior medical or surgical history. Physical examination revealed lower abdominal tenderness in addition to swelling and tenderness in the left inguinal region (Figure 1). The right testis was found in the scrotum during the genital examination but the left testis did not find. Since birth, no testicular examination has been performed for the boy.

## 3 DIFFERENTIAL DIAGNOSIS, INVESTIGATIONS, AND TREATMENT

**FIGURE 2:**
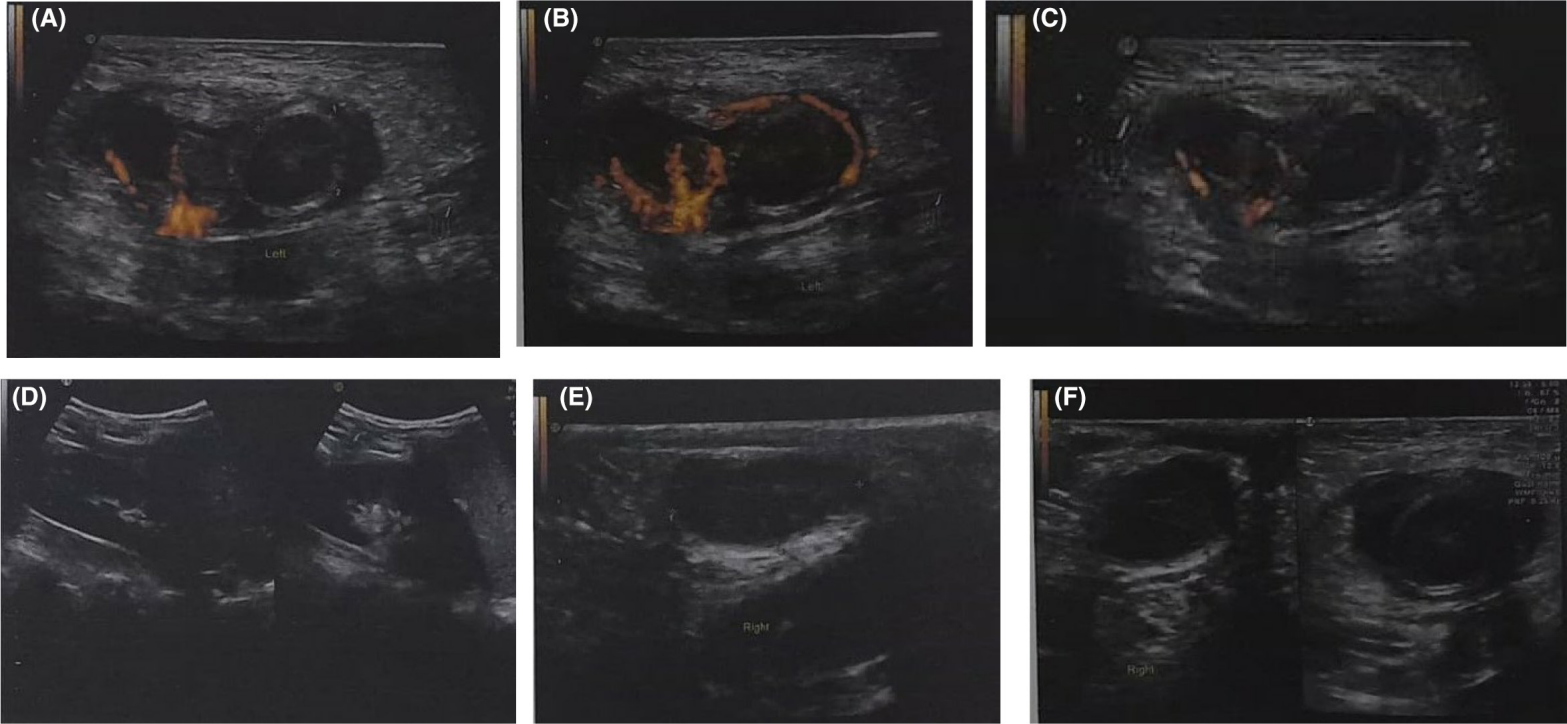
(A–C) The Doppler color scan demonstrated that the left testis in the inguinal area had a hyperechoic pattern, no blood flow, and excess fluid around it, and the left epididymis had a high blood flow and a hypoechoic pattern. (D, E) These figures demonstrate the right testis and epididymis by ultrasonography. (F) This figure demonstrates the difference between right and left testis.

**FIGURE 3:**
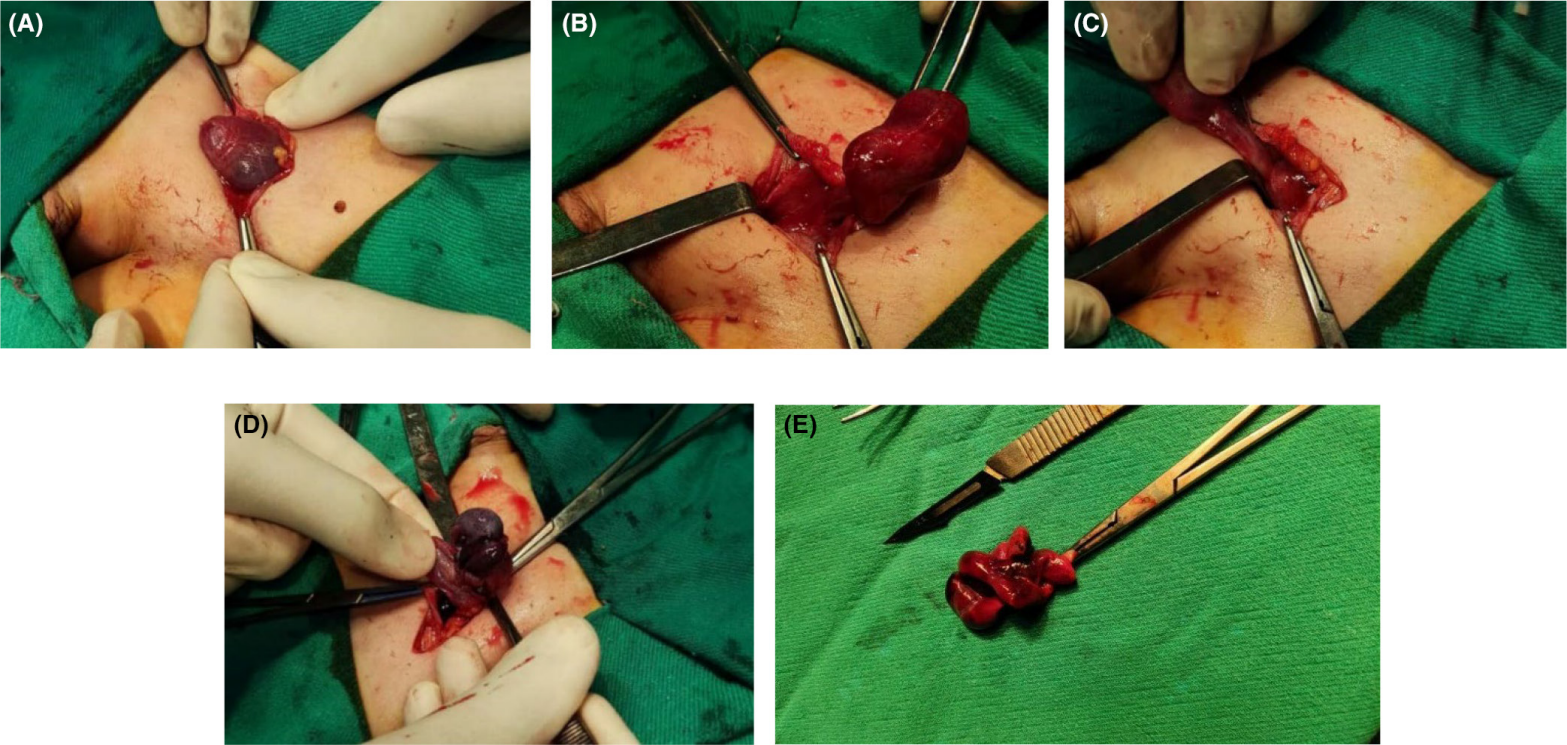
(A–C) Torsion testicle extraction following left inguinal incision. (D) Examining and separating the testicle from adjacent tissue. (E) Orchidectomy.

A complete blood count (CBC) showed elevated white blood cells (WBCs), particularly granular cells, which were 10.4 × 10^3^/μl, and other laboratory test results were normal. Ultrasonography and color Doppler demonstrated that the left testis did not exist and was found in the left inguinal region, measuring (15.2 × 12) mm, and had no blood flow in the testis and hypoechoic pattern. The right testis measured (18.8 × 9.1) mm was in the scrotum, and had significant blood flow and moderate echogenic pattern (Figure 2). The initial diagnosis was torsion of the undescended left testis. Under general anesthesia, a left inguinal incision was performed, revealing extravaginal testicular torsion (two round) inside the external inguinal ring, accompanied by testicular gangrene, with free fluid adhering to the adjacent tissue. After testicular examination, an orchidectomy was performed (Figure 3, Video S1).

## 4 OUTCOME AND FOLLOW‐UP

The patient was discharged in good condition the next day following surgery.

## 5 DISCUSSION

Undescended testicle, or cryptorchidism, is a definition used to describe a testicle that remains in its development trajectory rather than in the scrotum.^1^ Delasiauve described the first case of undescended testicular torsion in 1840. An undescended testicle can be found in the upper scrotum, superficial inguinal pouch, inguinal canal, or abdomen. Approximately 70% of undescended testicle cases are palpable. Approximately 30% of the nonpalpable testes are located in the inguinal‐scrotal region, 55% are intra‐abdominal, and 15% are absent or disappeared.^5^ The incidence of undescended testicles is 0.8%–1% in adults and children older than 1 year, with approximately 20% of these testes found intra‐abdominally.^4^ Researchers currently have no idea of the incidence or relative risk of torsion in an undescended testicle. According to many studies, torsion is more likely to occur in undescended testicle than in testes that have completely descended. One study suggested that the risk of torsion in cryptorchid testis is 10 times higher.^5^ It is unknown what pathophysiology contributes to the increased risk; some hypothesize that this difference is related to the ability of the spermatic cord to fully extend, while others propose that the heavier weight of the undescended testicle is the reason for torsion. In descended testes, torsion is correlated with the patency of the vaginal process. Another hypothesis is that improper contraction or spasm of the cremasteric muscle due to partial closure of the patency of the vaginal process, the warm inguinal area of the lower abdomen, or intra‐abdominal debris may prevent the undescended testes from migrating and causing testicular torsion.^2^, ^3^, ^4^ An inguinal testicular patient presented with inguinal pain, swelling, erythema, vomiting, abdominal pain, and inconsolable crying. The most significant consequences of cryptorchidism include a high incidence of testicular cancer and a high rate of infertility.^4^ Torsion of an undescended testicle can be difficult to diagnose because it may mimic other, more common disorders such as appendicitis, acute abdomen, and incarcerated hernia. Surgery is frequently delayed as a result of diagnostic confusion.^6^ It was feasible to perform color Doppler and ultrasonography and complete genitourinary examinations to confirm the diagnosis without avoiding critical surgical investigations.^1^, ^4^ The recommended approach for treating probable testicular torsion is rapid surgical exploration, regardless of where the testis is located.^1^ The recommended period for surgical exploration and orchidopexy is between 6 months and 18 months of age, as this is when the probability of a spontaneous descent becomes less likely.^7^ Orchiectomy is recommended if testicular necrosis has been observed and if there is no blood flow.^8^ In our case, we presented a 7‐year‐old child who had undescended testicle torsion in inguinal region, which is uncommon in this age range. There has not been any urogenital examination since the patient's birth due to poverty and neglect. The patient's symptoms were confirmed by ultrasound, which confirmed that the left testicle was torsion and located in the inguinal area. Additionally, color Doppler imaging showed no blood flow inside the testis. Surgical exploration revealed extravaginal testicular torsion, and testicular gangrene. An orchiectomy is done with the parent's consent. After the orchiectomy, the patient recovered well.

## 6 CONCLUSION

This case study underscores the importance of early detection and management of undescended testis to prevent serious complications. There have been very few reported cases of undescended testicle torsion in the inguinal region, and older age. Health care providers should be vigilant in performing thorough urogenital examinations in pediatric patients, particularly those from underserved communities. Further education and awareness efforts are needed to ensure timely intervention for undescended testis to improve outcomes for affected boys.

## AUTHOR CONTRIBUTIONS

**Yousef Alsaffaf:** Writing – original draft; writing – review and editing. **Hazem Arab:** Writing – original draft; writing – review and editing. **Jaafar Jaafar:** Writing – review and editing. **Mohammad Shehadeh:** Supervision.

## FUNDING INFORMATION

No funding is required.

## ETHICS STATEMENT

Not applicable because all data belong to the authors of this article.

## CONSENT

Written informed consent was obtained from the patient to publish this report in accordance with the journal's patient consent policy.

## Supporting information


Video S1.

## Data Availability

The data that support the findings of this study are available from the corresponding author upon reasonable request.
