# Supplementary material for: A 7‐year‐old with extravaginal torsion of an undescended testicle in the left inguinal region: The first case report from Syria
Source: Clin Case Rep. 2024 May 27;12(6):e9013. doi: 10.1002/ccr3.9013 (PMC11130230; doi:10.1002/ccr3.9013)
Supplement: Supplementary file 1 — Video S1. [file CCR3-12-e9013-s001.zip › ccr39013-sup-0002-VideoS1.docx]

**Video S1.** Intra-operation video demonstrates testicular torsion in the inguinal region within the external inguinal ring.
